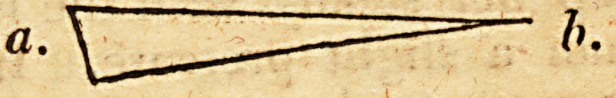# Case of Diseased Bladder, and Case of Divided Larynx

**Published:** 1804-01-01

**Authors:** I. Roche

**Affiliations:** Surgeon to the Third Regiment of Light Dragoons


CASE OF DISEASED BLADDER, *and* CASE OF DIVIDED LARYNX; *communicated by* MR. I. ROCHE, *Surgeon to the Third Regiment of Light Dragoons.*

THE diseases to which the bladder is liable, are various, sometimes giving way with rapidity, oftener extremely obstinate, and in some cases fatal; none, however, appear more distressing than incontinence of urine, from whatever cause it may have originated. The following case is worth attention from the celerity with which it yielded to the treatment treatment described, after its obstinate resistance to so many powerful agents previously employed.

William Reny, almost on his first essay on horsemanship, struck the lower part of his belly with much violence against the pummel of his saddle; the immediate consequences were external inflammation, and a constant flow of urine, immediately on its deposition, from the ureters. The various remedies generally suggested on such occasions were in vain exhibited. Blood-letting, cold applications, blisters, the internal use of tincture of cantharides, pressure, &c. afforded no relief, and he rather lost than gained ground. His stomach, heretofore good, was now much debilitated, his pulse quick and small, the abdomen painful and hard, and his penis in an indurated state of enlargement; add to these the distress of constant micturition, and the case will be allowed to have been unpromising. Having frequently observed the effect of counter irritation by producing a metastasis, I resolved to try the effect of rubbing over the abdomen the following ointment.

℞. Unguenti hydrargyri fortioris unciam, camphoræ solutæ paucis guttis spiritus vini rectificati drachmam dimidiam, M. et fricatur interim scrupulum secundis horis regioni abdominis.

I did not however wholly rely on this. In order to stimulate the sphincter vesicæ I introduced a middle sized catheter, as well as I could judge so far, as, that the perforations on its end would reach the entrance of the bladder. I then poured about thirty drops of tincture of opium, through the tube, which arriving near its end, emerged on every side, by which means I had reason to conclude the vis insita of the sphincter was roused into action. This was not attended with much pain, and was thrice repeated. After each rubbing of the ointment, I caused the abdomen to be firmly, yet not too tightly, swathed with a flannel roller; a practice I have found of much service in many diseases of the abdominal viscera. Pursuing the plan detailed, I had the satisfaction of inducing in two days a regular flow of urine completely under command; a soreness of the mouth had previously taken place, the camphor having quickened the action of the mercury. The patient has suffered no other inconvenience since, than a slight soreness through the course of the urethra, which, from the means employed, might very reasonably have been expected.

The second case is no further worth notice, than that it tends to prove, that a part of the thyroid cartilage may be removed without inconvenience, or injury to the voice.

John Benson, a strong muscular man, in a moment of insanity, attempting suicide, cut his throat in four places. I saw him almost on the instant, and found him bleeding profusely and labouring under irritation of the stomach, which consequently caused difficulty, and some loss of time. After the vomiting had in some degree ceased, I was able to make a minute inspection of the wounds. I found three of little consequence, the fourth was large and deep, the razor was twice drawn through this wound, and as often through the inferior part of the thyroid cartilage, which presented a sharp angular point, thus:

*a.*  *b.*

The point *b,* was directed outwards, it was therefore evident I could not close the wound, and leave it so situated, as it would have acted as an extraneous body, of course a cause of constant irritation; I dissected it off at *a,* leaving an apparent opening into the larynx, I brought, in making the interrupted suture, as much of the muscle and cellular membrane as possible over the opening, and inserted the needle as deep as I could without touching the cartilage; by which, I conceived, I guarded in some measure against its loss. From the first dressing no complaint whatever occurred; in one fortnight he was perfectly recovered; and it would not seem, that the least inconvenience has arisen from the loss of cartilage, a part apparently so necessary for the purposes of voice, inspiration, *&c.*

## Figures and Tables

**Figure f1:**